# Bioprospecting of four *Beauveria bassiana* strains and their potential as biological control agents for *Anastrepha ludens* Loew 1873 (Diptera: Tephritidae)

**DOI:** 10.1371/journal.pone.0324441

**Published:** 2025-06-27

**Authors:** Norberto A. Angel-Ruiz, Inés Zavala-Izquierdo, Diana Pérez-Staples, Francisco Díaz-Fleisher, Antonio Andrade-Torres, Griselda K. Guillén-Navarro, Pablo Colunga-Salas

**Affiliations:** 1 Instituto de Biotecnología y Ecología Aplicada, Universidad Veracruzana, Xalapa-Enríquez, Veracruz, México; 2 Departamento de Biotecnología Ambiental, El Colegio de la Frontera Sur (ECOSUR), Tapachula, Chiapas, México; National Institute of Agricultural Research—INRA, MOROCCO

## Abstract

*Anastrepha ludens* (Loew) is a pest of major importance on mango and orange crops. The use of biological control agents, including entomopathogenic fungi (EPF), has been widely studied. However, one problem with the use of EPF is that the efficacy of the strains varies with environmental conditions, and thus the use of native strains is suggested. Therefore, the objective of the present study was to bioprospect *Beauveria bassiana* strains from Veracruz, Mexico and determine their potential as biological control agents for *A. ludens*. Four strains isolated from infected insects were used to calculate conidium viability percentage, growth rate mycelia, and conidium production in three different media: with rice, with empty *A. ludens* pupae, and with PDA. The median lethal time (LT_50_) and median lethal concentration (LC_50_) of these strains were also calculated in *A. ludens* adults exposed to concentrations of 10^5^, 10^6^, and 10^7^ conidia/ml. The viability percentage ranged between 88%−98%, and the growth rate was higher in the rice media, with a value of 2.63 mm/day. However, conidium production was higher in the PDA and *A. ludens* pupae media, with values of 1.18x10^8^ and 7.83x10^7^ conidia/ml, respectively. At the highest concentration, the four strains caused mortality above 80%, and at the lowest concentration, only one strain caused mortality above 50%. The lowest LT_50_ occurred on day 5.51 at the highest concentration. The present study expands our knowledge on the effect of *B. bassiana* strains on *A. ludens*. In conclusion, the four strains used showed optimal levels for their potential use as biological control agents against *A. ludens*.

## Introduction

Entomopathogenic fungi (EPF) are widely distributed worldwide and, since the past century, have garnered interest in agriculture for their use in biological control due to their ability to infect arthropods [1,2]. They are facultative parasites able to cause pathogenesis exclusively in arthropods [3]. They are usually found in the environment in the form of conidia, which is the structure that initiates the infection cycle in the host [4,5]. Most of these fungi mainly belong to the phylum Ascomycota and to the genera *Lecanicillium*, *Metarhizium,* and *Beauveria*, which are characterized by being generalists, since they can parasitize different insect species [3–5].

The genus *Beauveria* has the capacity to infect over 700 species and is thus widely used as a biopesticide [6,7]. One of the most notable species of this genus is *Beauveria bassiana*, which is widely distributed worldwide, with records in soils of temperate zones, such as bogs, and dry zones, such as dunes [8,9]. The use of *B. bassiana* as a biological control agent has been successful against pests of economic importance worldwide, such as the coffee berry borer (*Hypothenemus hampei*) in India [10] or the silver-leaf white-fly (*Bemisia argentifolii*) under laboratory conditions in the United States [11]. Pathogenicity by this fungus has also been determined in fruit flies of the genera *Zeugodacus*, *Bactrocera*, *Ceratitis*, and *Anastrepha* [12–15].

One pest of major importance on citrus and mango crops in Mexico is the Mexican fruit fly, *Anastrepha ludens* [16]. This pest parasitizes the fruits in the larval stage, feeding on the pulp and causing the fruit to drop, which results in direct loss of the product [17]. This pest is currently controlled using integrated pest management (IPM), which has been proven to be compatible with the application of EPF, generating synergistic effects [18,19]. Even though over 30 strains of *B. bassiana* have been isolated in Mexico, very few have been tested against *A. ludens* [20–23].

Existing reports on the use of EPF against *A. ludens* in Mexico have involved strains from states where *A. ludens* is present. These studies have shown that the strains are effective, causing mortality of up to 90%, mainly in the adult stage [15,24,25]. They have also shown that the use of native EPF strains may increase the efficiency in the control of *A. ludens* populations, since strains adapted to their habitats have been reported to be more tolerant and efficient compared to non-native strains [26,27]. Thus, studying native strains of fungal species could potentially reveal more effective biological control agents for specific tropical areas and pest populations. Given the economic problem that *A. ludens* represents in Veracruz and the limited information about the pathogenicity of *B. bassiana* strains native to Veracruz, the objective of the present study was to bioprospect four *B. bassiana* strains isolated in Veracruz and determine their potential as biological control agents for *A. ludens.*

## Methods

### Isolation of strains

**Fig 1:**
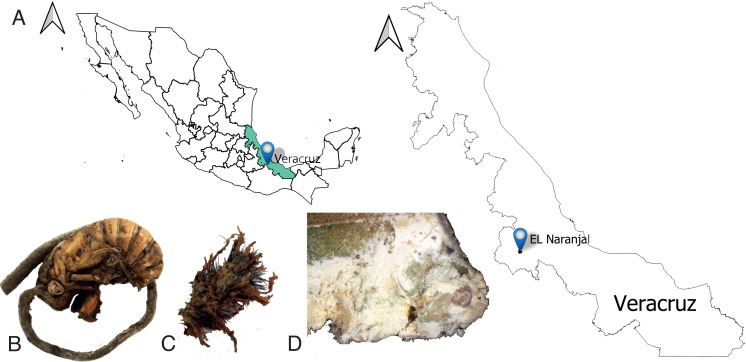
Sampling and insect collection site. A: Site of origin of the isolated *Beauveria bassiana* strains. B-D: Carcasses of insects collected in the Conservation Management Unit “Tequecholapa”; photos were taken by Norberto Angel-Ruiz. The map was created with the open software QGIS V3.40.2. All shapefiles used are freely available at https://www.inegi.org.mx/app/mapas/.

Convenience sampling was conducted from December 2023 to February 2024 by collecting insect carcasses with external presence of mycosis in the Conservation Management Unit (UMA, Spanish acronym) “Estación Ambiental Tequecholapa” in the municipality of Naranjal, Veracruz, Mexico (80°49’ N; 96°58’ W), located at an altitude of 740 masl (Fig 1A), and with presence of forest. Morphological or molecular identification of the hosts was not possible due to the high level of deterioration of the insects and the presence of fungi.

The fungi were isolated from the collected insects (Fig 1B–1D) in a potato dextrose agar (PDA) culture medium added with streptomycin at 1g/ lL in sterile Petri dishes of 90x15 mm. Small insect fragments were inoculated and incubated by triplicate on culture media for seven days at 27°C. The fungal colonies were inoculated in a new Petri dish with PDA medium by triplicate every three and seven days at 27°C until pure cultures were obtained.

The entomopathogenic capacity of 11 of the 46 isolates obtained was confirmed with a pilot test infecting fruit flies. Pathogenicity was confirmed by the presence or absence of the fungus on the carcasses of the individuals tested (S1 Table). Only the 11 entomopathogenic isolates were used for the viability and pathogenicity experiments.

### Conidium viability

Each strain was diluted to a concentration of 10^6^ conidia/ml using distilled water with 0.1% Tween 20. The number of conidia was calculated using a Neubauer chamber and the following equation:


$$\frac{N}{ncp}*\frac{cp}{1}*\frac{1cp}{L*A*P{mm}^{3}}*\frac{100{mm}^{3}}{1\;{cm}^{3}}*\frac{1{cm}^{3}}{1ml}*FD$$

where *N*: total number of conidia, *ncp*: number of small squares counted, *cp*: number of small squares in a big square, *L*: length of the big square, *A*: width of the big square, *P*: depth of the big square, *FD*: dilution factor.

Subsequently, to calculate conidium viability, aliquots from each dilution mentioned above were placed on sterile microscope slides with PDA and incubated at 27°C for 22 h, with six replicates per strain, following the methodology by Lopes et al. [28]. At 22 h, trypan blue was added to stop the germination of conidia, which were considered viable when they formed a germ tube larger than the conidium diameter [29]. The germination percentage of the first 100 conidia observed was obtained.

To evaluate if there were significant differences in viability between strains, the data were arcsine square root transformed and analyzed with a one-way ANOVA and a Tukey-HSD post hoc test in R 4.3.2 [30]. The results were used to select four representative strains for the subsequent analyses.

### Morphological and molecular identification of the fungus

Solid culture media prepared with PDA (Millipore Corporation, Damrstadt, Germany), rice extract, and extract from empty *A. ludens* pupae were used for morphological identification of the strains B1, B3, B4, and B10, which were those selected from the viability tests, using monosporic cultures. In general, the rice and pupal extracts were prepared at a concentration of 20 g per liter of water, 15 g of agar-agar per liter of the extract previously filtered with a 0.1-mm sieve. Finally, the media were sterilized in an autoclave for 15 min at 121°C.

Morphological identification was performed by describing the type of growth, coloration, elevation, and shape of the mycelia following the methodology by Cortez-Madrigal et al. [31]. The microscopic identification was made by measuring the mean size of the conidia (starting from the first 30 visible conidia) at 100X magnification using a Motic BA-410 microscope.

The molecular identification was made using three monosporic cultures in PDA media of each of the four selected strains, following the inoculation and incubation conditions previously described. On the fourth day of growth, the cultures were sent to the Genomic Services Laboratory of CINVESTAV Irapuato for the amplification and sequencing of four different markers. The internal transcribed spacer 1 (ITS1) and the 5.8S ribosomal RNA were partially sequenced, the internal transcribed spacer 2 (ITS2), and a fragment of the large subunit ribosomal RNA were completely sequenced. The amplifications were made with the oligonucleotides ITS1 (5- ´TCCGTAGGTGAACCTGCGG-3 ´) and ITS4 (5 ´-TCCTCCGCTTATTGATATGC-3 ´) [https://portal.cinvestav.mx/uga-langebio/investigacion/servicios/laboratorio-de-servicios-gen243micos-1]. This region was chosen because of its high utility and widespread use in the identification of fungal species [32,33].

The obtained sequences were corroborated with the BLASTn algorithm [https://blast.ncbi.nlm.nih.gov/Blast.cgi]. Subsequently, the sequences were manually edited using FinchTV 1.5 (Geospiza, Inc., Seattle, WA, U.S.A.). Multiple alignment was performed in AliView V. 1.28 [34] with the algorithm MUSCLE [35] using sequences of different species of the genus *Beauveria* available in GenBank, including a sequence of *Metarhizium anisopliae* as an external group.

The best partition scheme (considering the codons only in the coding regions 18S, 5.8S, and 28S) and substitution model for the concatenated data were calculated in ModelFinder [36]. The partition scheme and substitution model were used to infer the phylogenetic relationships by maximum likelihood in IQ-TREE [37] through a full search. Branch support was evaluated with 1,000 non-parametric bootstrap replicates.

### Mycelial growth and sporulation tests

Mycelial growth was evaluated using monosporic cultures of each strain, with three replicates per culture medium (rice, pupae, and PDA), following the methodology described above. All the media were incubated at 27°C for 15 days. Mycelial growth per strain and culture medium was observed and recorded daily throughout the incubation period, including a description of changes in morphology and coloration. Changes in growth were quantified with a millimeter ruler by measuring the radius of the mycelium. Mycelial growth was compared by day and culture medium using a generalized linear model (GLM) with a Poisson distribution and a log link function in R.

Spore counts per culture medium (rice, pupae, and PDA) and strain were performed using monosporic cultures on day 15 after inoculation. The conidia were obtained by scraping the surface of each culture and suspending it in a 0.1% Tween 20 solution with distilled water by shaking until the dilution was homogeneous. Subsequently, 10 µl were taken from each solution and placed on a Neubauer chamber to calculate the number of conidia using the equation mentioned above. The obtained data were log transformed and analyzed with a two-way ANOVA and a Tukey post hoc test in R.

### Pathogenicity tests

The hosts used were sterile *A. ludens* adults reared in the MOSCAFRUT facility, Mexico Domínguez et al. [38]. Sterile pupae were flown to INBIOTECA, where flies were kept at 26.5°C and provided with sugar and hydrolyzed protein (3:1 ratio) and water. Experiments were carried out with adults that were three to four days old.

To evaluate the pathogenicity of the strains, a two-factor experimental design with five replicates per treatment was used, where the factors were strain and conidium concentration. The flies were exposed to the fungus by immersing them for 30 s in 0.1% Tween 20 solutions at concentrations of 10^5^, 10^6^, and 10^7^ conidia/ml, which were obtained from 15-day PDA cultures and counted with a Neubauer chamber following the procedures described above. Five replicates per treatment (10^5^, 10^6^, and 10^7^ conidia/ml) were performed with 100 flies (50♀ and 50♂) each, including a negative control that consisted of a sterile 0.1% Tween 20 solution. Each treatment was placed in an entomological cage with water containers and diet. The cages were kept under environmental conditions for 12 days to calculate mortality, LC_50_, and LT_50_. The mean daily temperature recorded during the assay was 25.3°C, with a maximum of 37.2°C and a minimum of 18.5°C.

Daily mortality was quantified per treatment and replicate. All dead specimens were removed daily and individually sterilized by a 30-s immersion in a 0.01% NaClO dilution followed by a 30-s immersion in distilled water, a third 30-s immersion in 70% alcohol, and two final 30-s immersions in distilled water. The specimens were dried with paper towels between immersions. Finally, they were individually incubated in humidity chambers at 27°C in complete darkness for three days to check for mortality caused by EPF infection. None of the dead flies in the negative control showed mycelial growth. The data were analyzed to determine the mortality rate in each treatment using Kaplan-Meier survival curves and a proportional hazards analysis with the R package *survival.* The LT50 was also calculated using the R package *ecotox* [39,40].

## Results

### Isolation and conidium viability test

**Fig 2:**
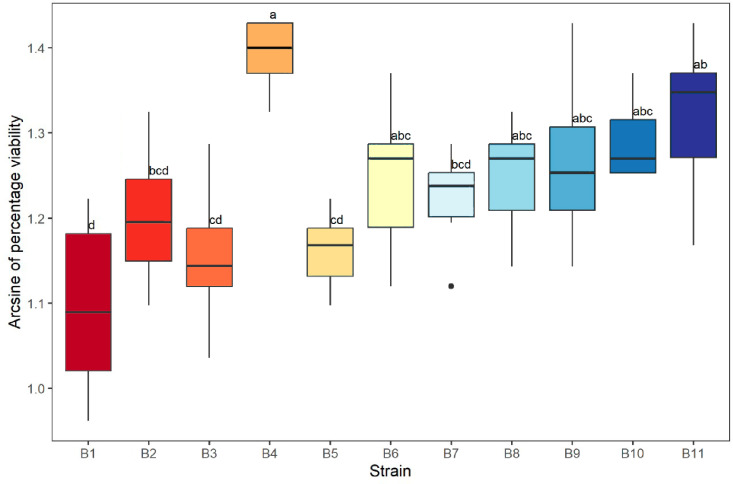
Conidium viability of each strain of entomopathogenic fungi isolated. Letters indicate significant differences (F_[10,55]_ = 6.8, P < 0.001).

Ten insects were collected inside the Conservation Management Unit (UMA acronym in Spanish), from which 44 fungal strains were isolated, 33 (75%) of which were saprophytic and 11 (25%) were entomopathogenic (S1 Table). There were significant differences (F _[10,55]_ = 6.8, P < 0.001; Fig 2) in viability at 22 h between the 11 entomopathogenic strains isolated. Strain B4 showed the highest viability, with 98.33% ± 0.81, followed by strain B11, with 96.5% ± 2.58, and strain B10, with 96% ± 1.26. The strains with the lowest viability were B5, with 91.66% ± 1.86, followed by B3, with 91% ± 3.37, and finally B1, with 88.5% ± 4.84 (Fig 2).

### Morphological and molecular identification

**Fig 3:**
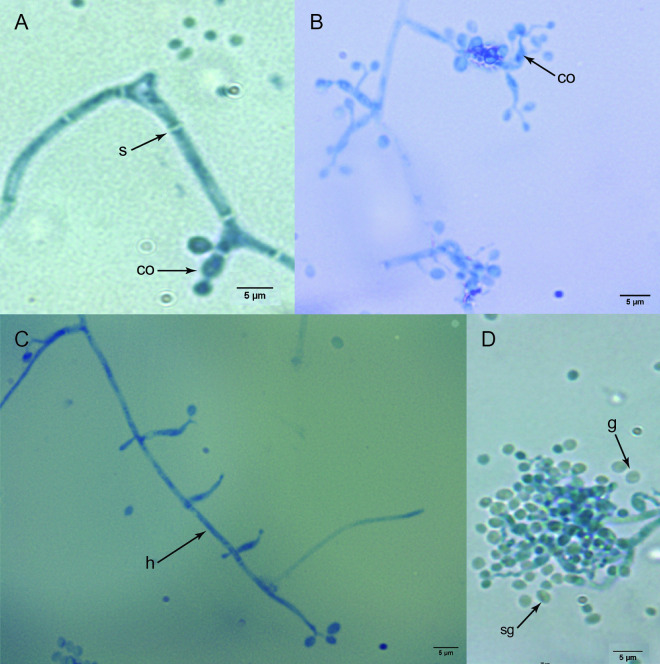
Microscopic structures of the *Beauveria bassiana* isolates. co: conidiophore, s: septa; h: hypha with conidiophores at different growth stages; g: globular and sg: semi-globular conidia.

In terms of morphology, the four isolates exhibited hyaline septate hyphae with flask-shaped basipetal conidiophores with a rachis with a zigzag appearance (Fig 3A, 3B). The conidiophores were arranged in clusters or individually (Fig 3A, 3C). The conidia had smooth walls, a globular or semi-globular shape (Fig 3D), and a mean size of 2.30 µm ± 0.20 in length and 1.97 µm ± 0.24 in width. Strain B3 had the largest conidia (2.54 µm ± 0.27 long and 2.29 µm ± 0.29 wide), while strain B10 had the smallest conidia (2.11 µm ± 0.20 long and 1.75 µm ± 0.17 wide).

**Fig 4:**
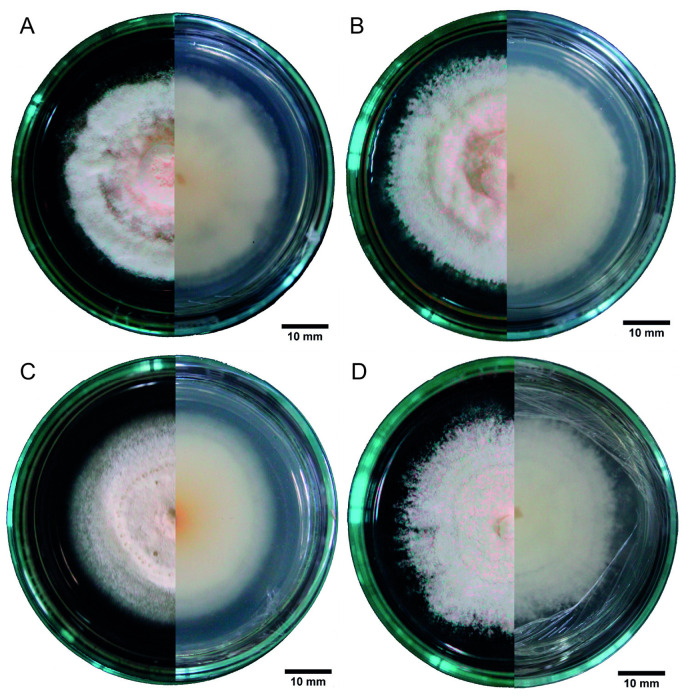
Mycelia of *Beauveria bassiana* in a PDA medium. In each panel, the left half of the image shows the top view, and the right half shows the bottom view of the Petri dish. A: strain B1, B: strain B3, C: strain B4, and D: strain B10.

Macroscopically, the four strains exhibited circular mycelia with concentric rings and smooth edges. In the PDA medium, all strains formed white, slightly yellowish mycelia at high densities on both the top and bottom surfaces (Fig 4). Strains B1 and B3 showed cottony, powdery aerial growth (Fig 4A, 4B), strains B4 and B10 showed a creeping-type growth (Fig 4C, 4D), while strain B4 was the only one with the presence of mycelial exudates on day 15 (Fig 4C).

**Fig 5:**
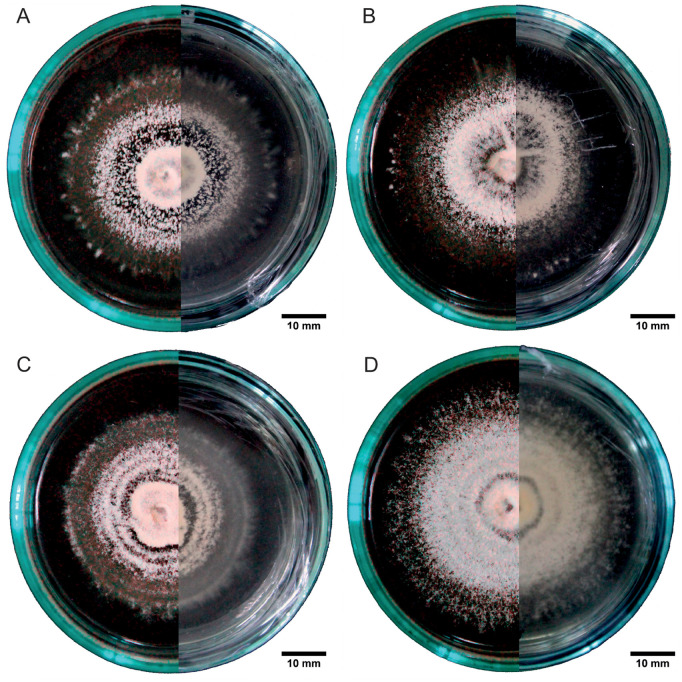
Mycelia of *Beauveria bassiana* in a pupae medium. In each panel, the left half of the image shows the top view, and the right half shows the bottom view of the Petri dish. A: strain B1, B: strain B3, C: strain B4, and D: strain B10.

**Fig 6:**
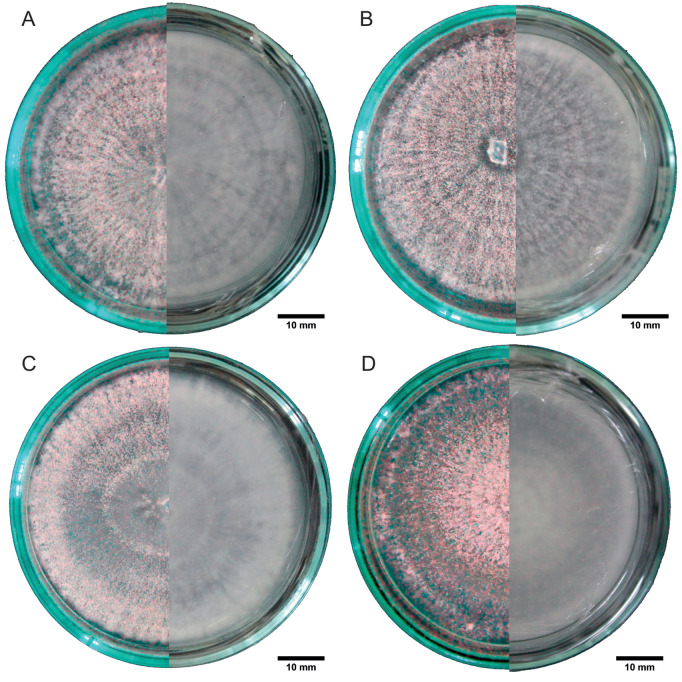
Mycelia of *Beauveria bassiana* in a rice medium. In each panel, the left half of the image shows the top view, and the right half shows the bottom view of the Petri dish. A: strain B1, B: strain B3, C: strain B4, and D: strain B10.

In the medium with pupae extract, the mycelia exhibited zonation (concentric bands with segments of different texture) and occurred at low densities (Fig 5A–5D). All the mycelia generally showed a whitish coloration with hyaline edges on the top surface and a slightly yellowish coloration at the center on the bottom surface. Finally, in the medium with rice extract, the four strains produced mycelia with creeping-type growth, zonation, hair-like hyphae, powdery white rings, and translucent segments (Fig 6A–6D).

In molecular terms, the sequences of the strains B1, B3, B4, and B10 were 100% identical to each other (GenBank accession numbers: PQ505989, PQ505990, PQ505991, and PQ505992). The analysis with BLAST showed 100% identity, an E-value of 0.0, and 100% coverage with sequences of *B. bassiana* from India (MT635019 and MT111135), Egypt (MN710408), and Colombia (MN427871). In the final alignment of 560 bp, there were 385 (68.75%) invariant sites, 92 (16.43%) singleton sites, and 51 (9%) parsimony-informative sites.

**Fig 7:**
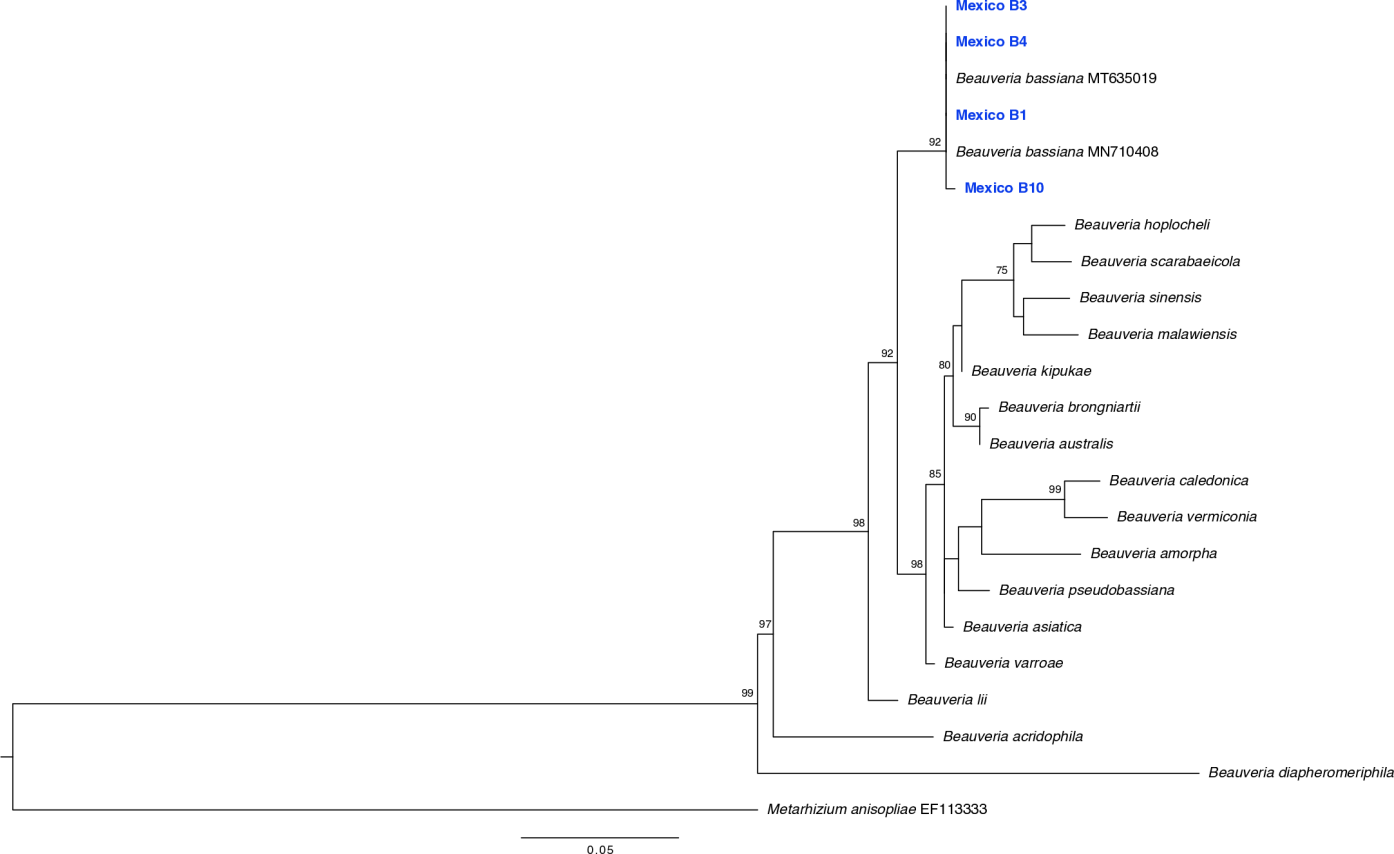
Phylogenetic reconstruction of an alignment of 549 bp (ITS1 + 5.8S rRNA + ITS2 + large subunit rRNA) of the genus *Beauveria.* The sequences obtained in the present study are indicated in blue.

Based on the best partition scheme and substitution model (18S, ITS1, and ITS2 = GTR + G; 5.8S and 28S = K2P), a phylogenetic reconstruction was obtained where the sequences from the present study were grouped with the reference sequences of *B. bassiana,* with a bootstrap value of 92%. This was the sister group of *Beaveria hoplocheli* + *Beauveria scarabaeicola *+ *Beauveria sinensis* + *Beauveria malawiensis* + *Beauveria kipukae* + *Beauveria brongniartii *+ *Beauveria australis *+ *Beauveria caledonica* + *Beauveria vermiconia* + *Beauveria amorpha* + *Beauveria pseudobassiana* + *Beauveria asiatica* + *Beauveria varroae*, which was differentiated with a branch support value of 92% (Fig 7).

### Mycelial growth and sporulation tests

**Fig 8:**
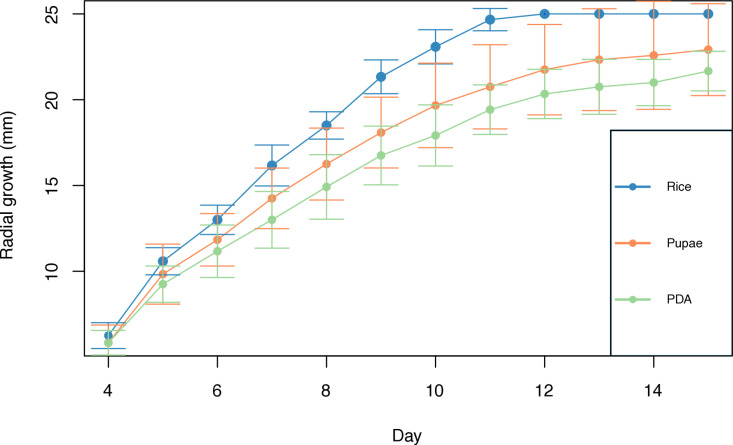
Mycelial growth curves from day 4 to day 15 in each culture medium. The mean values ± standard deviation calculated from the 3 replicates per strain are shown.

There was no effect of strain or the interaction strain-medium on mycelial growth on any of the days (Table 1). However, when comparing mycelial growth between culture media on each day, the rice medium showed a statistically higher growth compared to the other media on the ninth (χ2_[2,33]_ = 7.03, P = 0.02), tenth (χ2_[2,33]_ = 8.10, P = 0.01), eleventh (χ2_[2,33]_ = 8.14, P = 0.01), and twelfth day (χ2_[2,33]_ = 6.07, P = 0.04). On the last day, the mean growth was 22.91 mm ± 2.67 in the pupae medium, 21.66 mm ± 1.15 in the PDA medium, and 100% in the rice medium, without significant differences between media (χ2_[2,33]_ = 2.91, P = 0.23; Fig 8). Growth rate was 1.59 mm/day in the PDA medium, 1.81 mm/day in the pupae medium, and 2.63 mm/day in the rice medium, which was 1.65 times higher compared to that in the PDA medium.

**Table 1:** Generalized linear model (GLM) with a Poisson distribution of the effect of culture medium, strain, and their interaction on mycelial growth on each day.

		DF1	DF2	χ2	*P*
**Day 4**	Medium	2	33	0.23	0.89
	Strain	3	30	1.62	0.65
	medium:strain	6	24	0.64	0.99
**Day 5**	Medium	2	33	1.08	0.58
	Strain	3	30	1.72	0.63
	medium:strain	6	24	1.78	0.93
**Day 6**	Medium	2	33	1.71	0.42
	Strain	3	30	1.89	0.59
	medium:strain	6	24	1.16	0.97
**Day 7**	Medium	2	33	4.19	0.12
	Strain	3	30	2.04	0.56
	medium:strain	6	24	1.12	0.98
**Day 8**	Medium	2	33	4.72	0.09^*^
	Strain	3	30	2.62	0.45
	medium:strain	6	24	1.70	0.94
**Day 9**	Medium	2	33	7.03	0.02^*^
	Strain	3	30	2.13	0.54
	medium:strain	6	24	1.05	0.98
**Day 10**	Medium	2	33	8.1	0.01^*^
	Strain	3	30	2.86	0.41
	medium:strain	6	24	1.17	0.97
**Day 11**	Medium	2	33	8.14	0.01^*^
	Strain	3	30	1.59	0.66
	medium:strain	6	24	1.41	0.96
**Day 12**	Medium	2	33	6.07	0.04^*^
	Strain	3	30	1.92	0.58
	medium:strain	6	24	1.42	0.96
**Day 13**	Medium	2	33	4.84	0.08
	Strain	3	30	2.23	0.52
	medium:strain	6	24	1.52	0.95
**Day 14**	Medium	2	33	4.24	0.12
	Strain	3	30	2.08	0.55
	medium:strain	6	24	1.65	0.94
**Day 15**	Medium	2	33	2.91	0.23
	Strain	3	30	1.31	0.72
	medium:strain	6	24	1.18	0.97

Values marked with an asterisk (*) are statistically significant.

**Fig 9:**
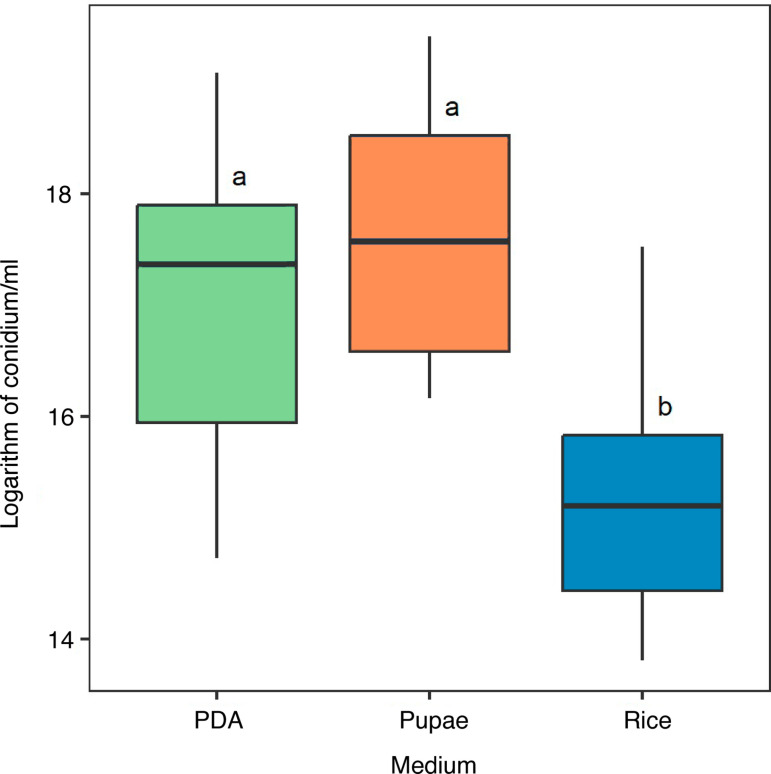
Concentration of conidia/ml in each culture medium. The combined data of the 3 replicates per strain are shown. Letters indicate significant differences (F_[2]_= 29.97, P < 0.001).

There were significant differences in the mean production of conidia/ml between media, where the highest concentration was observed in the pupae medium (1.18x10^8^ conidia/ml), followed by the PDA medium (7.83x10^7^ conidia/ml), and finally the rice medium (1.04x10^7^ conidia/ml), which was significantly lower than the other two (F_[2]_ = 29.97, P < 0.001; Fig 9).

### Pathogenicity tests

The Kaplan-Meier test revealed significant differences in survival in all treatments compared to the control (P < 0.001). All strains caused higher mortality at a concentration of 10^7^ conidia/ml on day 12 compared to the control (S1 Fig), where strain B10 caused the highest mortality percentage (97.4% ± 6.10), while the lowest mortality was caused by strain B3 on the same day (83.4% ± 6.38; Table 2; S1 Fig). The proportional hazards analysis showed a higher mortality associated with the strains compared to the control at the same conidium concentration (t_[4]_ = 1484; P < 0.001). The highest increase in mortality was caused by strain B4, with an increase of 2800% compared to the control (Z = 23.46; P < 0.001; S1 Fig). In contrast, strain B3 caused the lowest increase in mortality (1300%), although it was a significant increase compared to the control (Z = 18.27; P < 0.001; S1 Fig). Finally, strain B3 resulted in the highest LT_50_, which was 7.63 days, while strain B1 resulted in the lowest LT_50_, which was 5.51 days (Table 3).

**Table 2:** Mortality of *Anastrepha ludens* adults exposed to different *Beauveria bassiana* conidium concentrations.

	10^5^ conidia/ml				10^6^ conidia/ml				10^7^ conidia/ml			
**Strain**	**Day 7**	**Day 8**	**Day 9**	**Day 12**	**Day 7**	**Day 8**	**Day 9**	**Day 12**	**Day 7**	**Day 8**	**Day 9**	**Day 12**
**Control**	7.2 ± 2.6	7.4 ± 2.9	7.6 ± 2.9	9.4 ± 4.6	7.2 ± 2.6	7.4 ± 2.9	7.6 ± 2.9	9.4 ± 4.6	7.2 ± 2.6	7.4 ± 2.9	7.6 ± 2.9	9.4 ± 4.6
**B1**	15.6 ± 3.8	25.4 ± 3.5	29.8 ± 5.4	37.4 ± 5.	**50.2 ± 6.8**	71.8 ± 5.8	79.6 ± 7.9	90 ± 3.7	**63.2 ± 6.5**	83.4 ± 6.2	92.2 ± 3.5	96.8 ± 3
**B3**	18.8 ± 3.3	23.2 ± 4.3	28.4 ± 8.7	40.8 ± 14.6	42 ± 3	**55 ± 5.3**	64.8 ± 4.8	83.4 ± 2.7	40.4 ± 4.3	**53.2 ± 6.1**	61.6 ± 4.9	83.4 ± 6.4
**B4**	33.2 ± 8.5	45.8 ± 14.7	**51.6 ± 13.2**	62.8 ± 13.9	31.2 ± 17.7	**53.6 ± 2.9**	60.2 ± 1.5	71 ± 2.5	**70.8 ± 7.9**	85.8 ± 4.1	87 ± 3.5	95.6 ± 1.5
**B10**	18.8 ± 7.7	32 ± 14.7	37 ± 18.4	43.4 ± 17.4	39 ± 3.4	**61.4 ± 4.8**	69.8 ± 3.5	79.6 ± 3.7	**66.4 ± 9.8**	87.8 ± 7.4	93 ± 6	97.4 ± 6.1

All values are express as mean ± SD. Values in bold show the day on which mortality was equal or higher than 50%.

**Table 3:** Median lethal time of different *B. bassiana* strains at different concentrations.

Strain	Concentration (conidia/ml)	LT_50_	95% CI
**B1**	10^5^	17.82	15.83-20.68
	10^6^	6.40	5.79-7.02
	10^7^	5.51	4.46-6.51
**B3**	10^5^	16.27	14.13-19.76
	10^6^	7.66	5.77-10.41
	10^7^	7.63	6.91-8.49
**B4**	10^5^	9.76	8.73-11.28
	10^6^	8.52	7.79-9.45
	10^7^	5.78	3.07-8.11
**B10**	10^5^	11.81	10.90-13.13
	10^6^	7.71	6.58-9.08
	10^7^	6.01	5.64-6.37

The concentration of 10^6^ conidia/ml of all strains resulted in higher mortality compared to the control (S2 Fig), where the strains that produced the highest and lowest total mortality were B1 (90% ± 3.67) and B4 (71% ± 2.45), respectively (Table 2; S2 Fig). According to the proportional hazards analysis (t _[4]_ = 901.4; P < 0.001; S2 Fig), the most pathogenic strain was B3, which increased adult mortality by 1800% (Z = 20.45; P > 0.001; S2 Fig), and the least pathogenic strain was B4, which increased mortality by 1000% (Z = 16.29; P < 0.001; S2 Fig). Strain B1 resulted in an LT_50_ of 6.40 days, while the strains B3, B4, and B10 reached an LT_50_ between days 7 and 8 (Table 3).

Similarly, the lowest conidium concentration (10^5^ conidia/ml) of all strains resulted in higher mortality compared to the control (S3 Fig). The strains that produced the highest and lowest total mortality were B4 (62.8% ± 13.90) and B1 (37.4% ± 5.94), respectively (Table 2; S3 Fig). Strain B4 was also the only one that reached a value of LT_50_ before day 10 (TL_50_: 9.76; Table 3) and caused up to 795% higher mortality compared to the control (Z = 14.401; P > 0.001; S3 Fig).

## Discussion

To the best of our knowledge, the present study provides the first report of *B. bassiana* isolates obtained from the mountainous region of Veracruz, Mexico, which increases our knowledge of the effect of different strains of this fungal species on *A. ludens* in Mexico. The four strains evaluated showed high control levels against *A. ludens*. Even though the FAO/IAEA (2019) suggest an LT_50_ of 3 and 4 days for the use of EPF as biological control agents, the lowest LT_50_ in the present study occurred on day 5.51 and, although it exceeds the suggested value, it is within the period of sexual maturation of *A. ludens* adults, which has been reported to occur between days 10 and 15 after emergence in wild populations, and is considered the period were pests affect crops [41]. On the other hand, the use of EPF can enhance the control of these species by increasing the number of infected wild individuals when applied simultaneously with other IPM measures such as the Sterile Insect Technique [15,42].

### Bioprospecting of *B. bassiana*

The phylogenetic analysis of the four strains analyzed showed little variability among isolates at a global scale, which suggests that these regions (ITS1, ITS2, and 18S) are highly conserved in the genus *Beauveria* and confirms the utility of these molecular markers in the identification of fungi at a species levels [43,44]. *Beauveria bassiana* is currently recognized as a species complex [45], and thus the implementation of other markers, such as the nuclear intergenic region B (Bloc), may provide more information about the genetic diversity of *B. bassiana* and allow the evaluation of the relationship between the genetic diversity and place of origin of the strains, and this information will make it possible to determine if there are strains that could be potentially used more generally as biological control agents in temperate or tropical areas.

The genus *Beauveria* is morphologically identified by the size and shape of the conidia and conidiophores and the coloration of the mycelium [46]. The four isolates obtained in the present study exhibited conidia, conidiophores, and whitish mycelia consistent with the characteristics of the genus [47,48]. However, identification at the species level using these characters is difficult because different species of the genus share morphological similarities [46]. Therefore, future integrative taxonomy (morphology, phylogenetics, chemotaxonomy) studies are needed for an accurate delimitation of species within the *B. bassiana* complex [49].

The germination percentages obtained in the present study (93.92% ± 2.74) at 22 h are within the range of 47.8% to 100% previously reported for *B. bassiana* strains from Portugal and Indonesia at 24 h [50,51]. However, the obtained values are low compared to the germination percentages of isolates from Colombia, which were >95% at 20 h [52], or those observed in strains from Spain, India, United States, and Philippines, which were 100% at 16 h [53]. These differences may be due to biochemical changes that affect growth rate as a response to the amount of available nutrients, which can lead to variations in growth rate, as has been previously reported in *B. bassiana* [54–56].

The growth rates of the strains cultured in the media with pupae extract (1.81 mm/day) and PDA (1.59 mm/day) were lower than those reported for *B. bassiana* strains from Iran (2.99–4.59 mm/day) [55] and other species of entomopathogenic fungi such as *Isaria fumosoroseus* (2–4 mm/day) [57] and *M. anisopliae* (2.07–5.09 mm/day) [58]. In contrast, the results obtained with the rice medium (2.63 mm/day) are within the range of the reported values, which indicates the effectiveness of rice as a substrate for the growth and maintenance of fungal cultures, considering the low cost of production.

Even though the cultures in the rice medium grew faster, the production of conidia was significantly lower, which contrasts with the results from other studies where values of up to 3.94x10^12^ conidia/kg of rice have been reported [59–61]. These higher values may be due to the inoculation of the fungus directly on the rice grains, which would result in a better substrate contact area to volume ratio and therefore a higher spore production [62]. In the present study, we used a medium with rice extract where the area of contact with the substrate and nutrient availability may be reduced compared to inoculation directly on the rice grain [60,61]. This may explain the low yields obtained compared to the other media used.

The conidium concentration obtained in the isolates cultured in the PDA and pupae media was within the reported range and was even higher than those recorded in media prepared with cellulose (2.00x10^6^ and 3.24x10^7^ conidia/ml), glucose (5.27x10^7^ conidia/ml), and chitin peptone (6.7x10^6^, 6.5x10^6^, and 1.05x10^7^ conidia/ml) [55,63,64]. A higher conidium production in the pupae medium may be explained by the composition of the pupa. For example, some proteins constitute as much as 27.9% of pupae of *Lucilia cuprim*, and carbon compounds, such as chitin [65], may favor the development of *B. bassiana*. These results suggest that the implementation of substrates prepared with pupae may be an alternative for producing conidia at high concentrations. However, further studies are necessary to determine what proteins present in the exuviae of *A. ludens* favor the production of conidia.

### Pathogenicity tests

Overall, the mortality caused by the four strains was higher than 80%, and reached up to 97.4% at the highest concentration, and LT_50_ value of up to 5.51 days. These values are higher than those reported with strains from Brazil, where the LT_50_ was 3 days and the LC_50_ was 10^5^ conidia/ ml [15]. Nevertheless, our results are promising when compared to values reported with other Mexican strains of *B. bassiana* in *A. ludens* adults, with mortalities between 52.7% and 98% within a range of 6–12 days [15,25,66]. When comparing the mortality observed in the present study with that reported with a *B. bassiana* strain native to El Soconusco, Chiapas (south of Mexico), our results suggest a better performance of the strains from Veracruz, since the strain from Chiapas had an LT_50_ of 6–7 days at higher concentrations (10^9^ and 10^10^ conidia/ml) [25]. A study by de la Rosa et al. [15] reported mortalities between 82 and 98%, but the conidium concentrations were higher (10^8^) than those used in the present study.

Considering the above, the use of *B. bassiana* strains from the mountainous region of Veracruz, Mexico is a viable option for the control of *A. ludens*. The efficacy of these strains may be due to the similar climate and soil conditions between the place of origin of the strains and the regions in Veracruz where *A. ludens* is an important pest, and thus their application in the field may be even more effective than the application of non-native strains [67–70].

Finally, given that the production of viable infective propagules of EPF is affected by the strain’s origin, the culture medium, and the storage environment [27], it is important to consider conducting additional studies to evaluate the use of *A. ludens* pupae to enrich culture media for the production of EPF. This may allow the production of larger fungal colonies, a higher number of viable conidia and/or conidia with higher infectivity compared to those produced using artificial substrates, as has been demonstrated by using cricket powder to enrich culture media prepared with agricultural waste to grow *B. bassiana* [71].

## Conclusion

In conclusion, the present study analyzed four strains of *B. bassiana* with similar mycelial growth and propagule production. Our findings suggest that *B. bassiana* is a species complex that should be addressed in the future, as this genetic diversity could probably explain the different infectivity rates in different hosts. On the other hand, controlling nutritional factors in the culture media may affect propagule production of *B. bassiana*. The pathogenicity of the fungal strains in *A. ludens* adults appears to be unrelated to conidia production. Even though the LT_50_ and LC_50_ from the fungal strains in this study were lower than those reported for other Mexican strains, their potentially use as biological control agents against *A. ludens* in the field in Veracruz, Mexico could be more effective compared to non-native strains of the region, due to their habitat adaptation. The dissemination of native strains through novel methods, such as the use of ants as vectors, could also offer an additional benefit to control this pest [72].

## Supporting information

S1 Table Fungal strains obtained from each insect collected in the Conservation Management Unit “Tequecholapa”. (DOCX)

S1 Fig Survival curves (A) and Hazard ratios (B) obtained with each strain in the pathogenicity test at a concentration of 10^7^ conidia/ml. (TIF)

S2 Fig Survival curves (A) and Hazard ratios (B) obtained with each strain in the pathogenicity test at a concentration of 10^6^ conidia/ml. (TIF)

S3 Fig Survival curves (A) and Hazard ratios (B) obtained with each strain in the pathogenicity test at a concentration of 10^5^ conidia/ml. (TIF)
