# Supplementary material for: Bioprospecting of four Beauveria bassiana strains and their potential as biological control agents for Anastrepha ludens Loew 1873 (Diptera: Tephritidae)
Source: PLoS One. 2025 Jun 27;20(6):e0324441. doi: 10.1371/journal.pone.0324441 (PMC12204472; doi:10.1371/journal.pone.0324441)
Supplement: S1 Table — (DOCX) [file pone.0324441.s001.docx]

**S1. Table.** Fungal strains obtained from each insect collected in the Conservation Management Unit “Tequecholapa”.

| Insect | ID | Strain name | Saprophytic/Entomopathogenic |
| --- | --- | --- | --- |
| 1 | B1 | BI1.1.Tqcl | Entomopathogenic |
|  | B13 | BI1.2.Tqcl | Saprophytic |
|  | B8 | BI1.3.Tqcl | Entomopathogenic |
| 2 | B12 | BI2.1.Tqcl | Saprophytic |
|  | B14 | BI2.2.Tqcl | Saprophytic |
|  | B2 | BI2.3.Tqcl | Entomopathogenic |
| 3 | B3 | BI3.1.Tqcl | Entomopathogenic |
| 4 | B15 | BI4.1.Tqcl | Saprophytic |
|  | B16 | BI4.2.Tqcl | Saprophytic |
|  | B4 | BI4.3.Tqcl | Entomopathogenic |
|  | B6 | BI4.4.Tqcl | Entomopathogenic |
|  | B9 | BI4.5.Tqcl | Entomopathogenic |
| 5 | B11 | BI5.1.Tqcl | Entomopathogenic |
|  | B17 | BI5.2.Tqcl | Saprophytic |
|  | B18 | BI5.3.Tqcl | Saprophytic |
|  | B19 | BI5.4.Tqcl | Saprophytic |
|  | B5 | BI5.5.Tqcl | Entomopathogenic |
| 6 | B20 | BI6.1.Tqcl | Saprophytic |
|  | B21 | BI6.2.Tqcl | Saprophytic |
|  | B22 | BI6.3.Tqcl | Saprophytic |
|  | B23 | BI6.4.Tqcl | Saprophytic |
|  | B24 | BI6.5.Tqcl | Saprophytic |
|  | B25 | BI6.6.Tqcl | Saprophytic |
| 7 | B26 | BI7.1.Tqcl | Saprophytic |
|  | B27 | BI7.2.Tqcl | Saprophytic |
|  | B28 | BI7.3.Tqcl | Saprophytic |
|  | B29 | BI7.4.Tqcl | Saprophytic |
|  | B30 | BI7.5.Tqcl | Saprophytic |
|  | B31 | BI7.6.Tqcl | Saprophytic |
|  | B32 | BI7.7.Tqcl | Saprophytic |
| 8 | B33 | BI8.1.Tqcl | Saprophytic |
|  | B34 | BI8.2.Tqcl | Saprophytic |
|  | B35 | BI8.3.Tqcl | Saprophytic |
|  | B36 | BI8.4.Tqcl | Saprophytic |
|  | B37 | BI8.5.Tqcl | Saprophytic |
| 9 | B38 | BI9.1.Tqcl | Saprophytic |
|  | B39 | BI9.2.Tqcl | Saprophytic |
|  | B40 | BI9.3.Tqcl | Saprophytic |
|  | B41 | BI9.4.Tqcl | Saprophytic |
| 10 | B10 | BI10.1.Tqcl | Entomopathogenic |
|  | B42 | BI10.2.Tqcl | Saprophytic |
|  | B43 | BI10.3.Tqcl | Saprophytic |
|  | B44 | BI10.4.Tqcl | Saprophytic |
|  | B7 | BI10.5.Tqcl | Entomopathogenic |
