# Supplementary figures and images for: Bioprospecting of four Beauveria bassiana strains and their potential as biological control agents for Anastrepha ludens Loew 1873 (Diptera: Tephritidae)
Source: PLoS One. 2025 Jun 27;20(6):e0324441. doi: 10.1371/journal.pone.0324441 (PMC12204472; doi:10.1371/journal.pone.0324441)

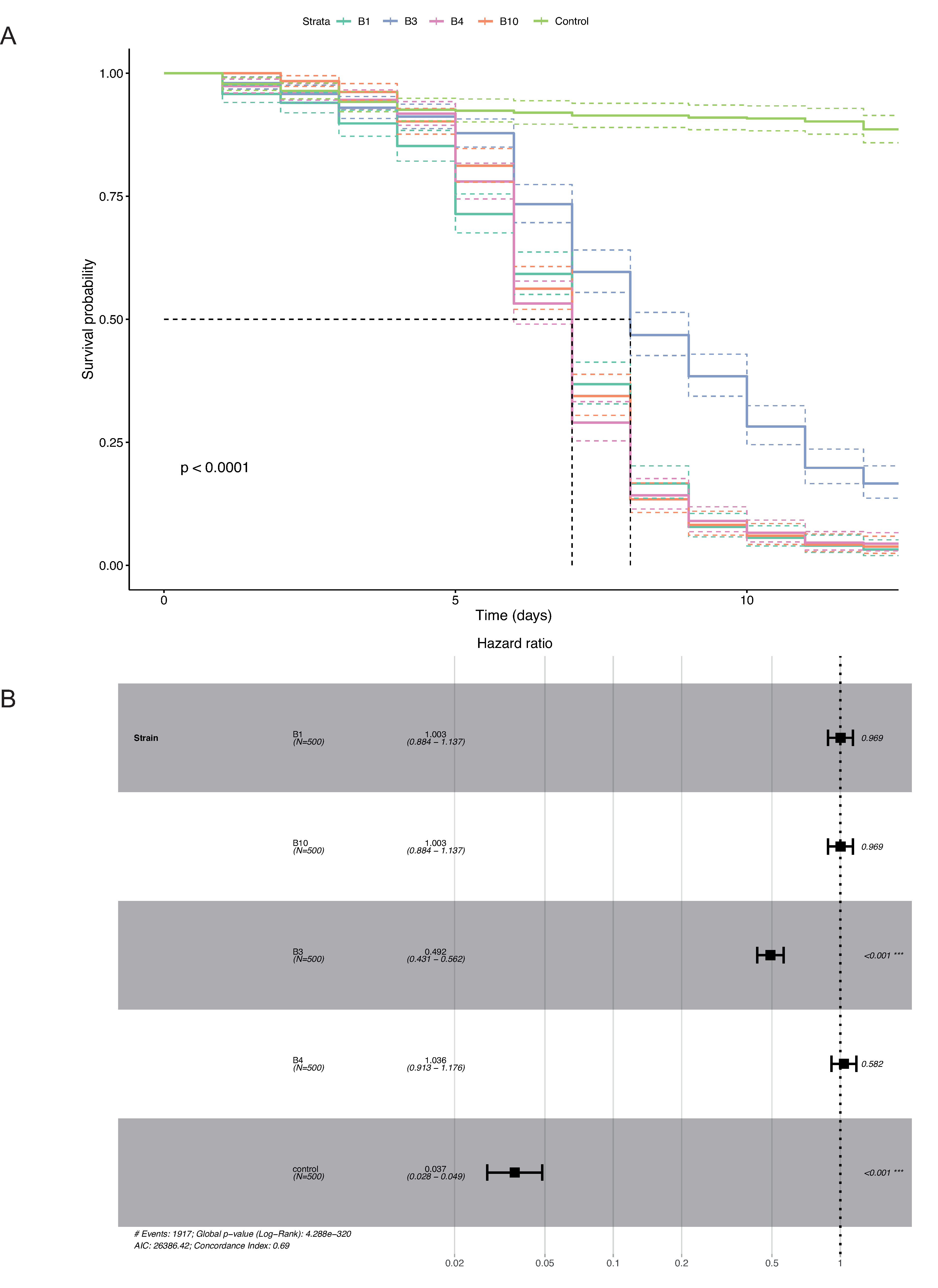

Supplement: S1 Fig — (TIF) [file pone.0324441.s002.tif]

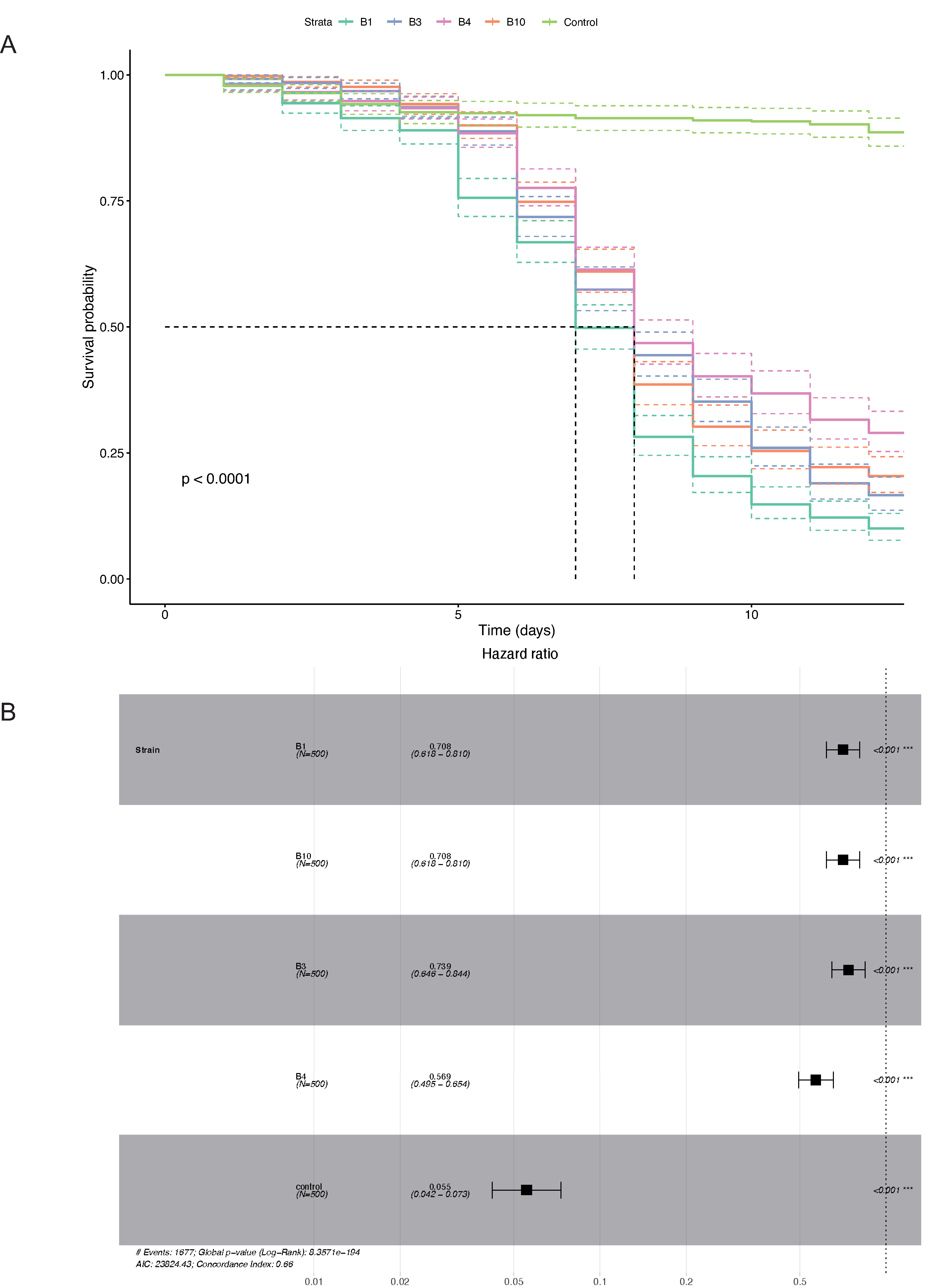

Supplement: S2 Fig — (TIF) [file pone.0324441.s003.tif]

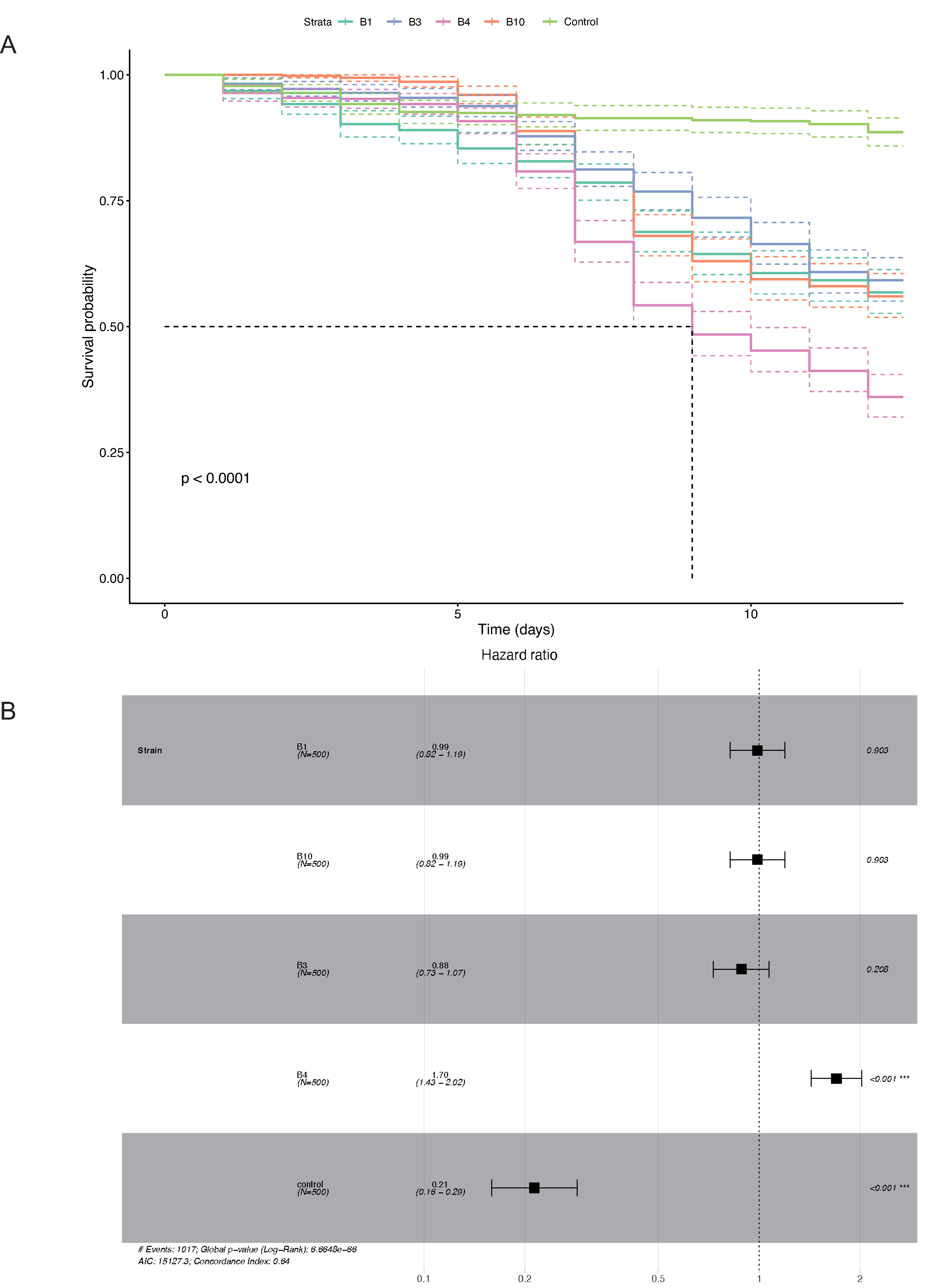

Supplement: S3 Fig — (TIF) [file pone.0324441.s004.tif]
